# Evidence for cultural differences in affect during mother–infant interactions

**DOI:** 10.1038/s41598-023-31907-y

**Published:** 2023-03-24

**Authors:** Miada Abu Salih, Maayan Abargil, Saja Badarneh, Nathalie klein Selle, Merav Irani, Shir Atzil

**Affiliations:** 1 Department of Psychology, The Hebrew University of Jerusalem, Mount Scopus, Jerusalem, Israel; 2 Criminology Department, Bar Ilan University, Ramat Gan, Israel

**Keywords:** Human behaviour, Psychology

## Abstract

Maternal care is considered a universal and even cross-species set of typical behaviors, which are necessary to determine the social development of children. In humans, most research on mother–infant bonding is based on Western cultures and conducted in European and American countries. Thus, it is still unknown which aspects of mother–infant behaviors are universal and which vary with culture. Here we test whether typical mother–infant behaviors of affect-communication and affect-regulation are equally represented during spontaneous interaction in Palestinian-Arab and Jewish cultures. 30 Palestinian-Arab and 43 Jewish mother–infant dyads were recruited and videotaped. Using AffectRegulation Coding System (ARCS), we behaviorally analyzed the second-by-second display of valence and arousal in each participant and calculated the dynamic patterns of affect co-regulation. The results show that Palestinian-Arab infants express more positive valence than Jewish infants and that Palestinian-Arab mothers express higher arousal compared to Jewish mothers. Moreover, we found culturally-distinct strategies to regulate the infant: increased arousal in Palestinian-Arab dyads and increased mutual affective match in Jewish dyads. Such cross-cultural differences in affect indicate that basic features of emotion that are often considered universal are differentially represented in different cultures. Affect communication and regulation patterns can be transmitted across generations in early-life socialization with caregivers.

## Introduction

Emotion regulation is the process of modulating the duration or amplitude of internal feeling states^1^ in an instrumental way^2^ for the service of self and social adaptation^3,4^. Emotion regulation is fundamental for health and well-being throughout the lifespan^5–7^. Deficits in emotion regulation underlie myriad forms of mental health adversities^8,9^ and contribute to deficits in relationships^10^, decision-making^11^, and job performance^12^. It is well known that emotion regulation develops over early childhood^6,13^ into adolescence^14^ and is thought to be scaffolded by caregivers’ socialization behaviors^15–19^. Although it is intuitive that parental care may promote infants’ emotion regulation, the behavioral mechanisms by which caregiver behavior translates into infant regulation is still relatively little understood.

Maternal care is considered a universal and even cross-species set of typical behaviors^20–23^, which are necessary to determine the social, cognitive, and emotion development of children^24–26^. Among the most typical and basic behaviors that mothers and infants spontaneously demonstrate are affect communication and affect regulation^27^. Affect communication and regulation are considered universal ingredients of mother–infant interactions^28–30^. However, the universality of affective communication in the mother–infant relationship requires further investigation, as the majority of research on the mother–infant relationship is based on Western, Educated, Industrialized, Rich, and Democratic (WEIRD) cultures, and conducted in European and American countries^25,26^.

Communication of affect is a core ingredient in mother–infant interaction^31,32^ and refers to the social communication of arousal and valence^33^. Since human infants are born helpless in maintaining their ongoing physiological demands, they depend on their caregivers for biological and psychological regulation and communicate their regulatory needs using affective cues^33^. Infants communicate negative changes in regulation (e.g., pain, hunger, frustration, or fatigue) by frowning, fussing, or crying. Infants communicate positive changes in regulation (e.g., soothing, joy and interest) by smiling, laughing, and engaging. Thus, infants’ affective signals bare information about their regulatory requirements, which is salient for caregivers. Accordingly, caregivers tend to be highly attentive to their infant's affective cues, which effectively elicit parental dopaminergic secretion^33^ and parental care behaviors^34^. This interactive process allows the parent to learn and predict the infant's needs to provide attuned care, which is defined here as *social regulation of allostasis*^35–37^. Affect communication is thus necessary for the social regulation of allostasis^15^. Moreover, parents regulate the infant affect via affective attunement, which supports infant development^35,38–40^, as through affective communication and affective attunement caregivers teach infants emotion concepts, self-regulation, and the value of phenomena in the environment^15,28,41^. Affect is thus a basic feature of the parent–infant relationship, that is central to infant survival and development^27,38,42–45^. Despite being a basic and necessary feature, it is still unknown which aspects of affect are universal and which vary with culture.

Amongst affect, cultural differences were found in a variety of mother–infant behaviors^46^. For example, African mothers talk less to their infants compared to mothers from the Far East, Europe, and South and North America^47^. Indian mothers and infants tend to vocalize simultaneously compared to French and American mothers and infants, who take turns in vocalizations^48^. Moreover, Lebanese infants show a faster vocal response to their mother compared to Western infants^49^. Cross-cultural differences were also found in attachment styles, as in western cultures the dominant insecure attachment style is avoidant, whereas in non-western cultures the dominant insecure attachment style is resistant^50^. Such cross-cultural differences were suggested to rely on different cultural values and beliefs about parenting^51^. The cultural differences in attachment are of particular interest, given the previously reported association between attachment and the development of affect regulation, which is necessary for resilience^52^. For example, securely attached children were reported to show increased affective communication and to modulate their emotional responses^53^. Thus, cross-cultural differences in attachment could be routed in cultural differences in affect communication.

In adults, while some studies refer to affect communication^54,55^ and affect attunement^56^ as universal, other studies show that affect communication varies between cultures^57^. For example, the understanding of certain facial motions as prototypical of a specific emotion is based on cultural knowledge^58,59^. Moreover, some facial expressions are conveyed and recognized only in specific cultures^60^. Studies also show cross-cultural variation in the intensity of emotion expression^61,62^. Like affect, the tendency to synchronize in attunement with a partner during interaction is mostly considered a universal phenomenon^63^. Yet, studies show cross-cultural variation in some aspects of interpersonal interactions^64^. For example, while in Western countries only positive interactions are considered synchronous^65,66^, in Japan negative interactions are perceived as synchronous as well^67^.

The Arab society is under-studied^68–70^ and Palestinian Arabs are particularly under-represented in scientific investigation across disciplines, including social, medical, and psychological research^71^, and particularly in the field of maternal bonding. Along with East-Asian cultures, Arab cultures are considered "Eastern" collectivist cultures^72,73^. Collectivist cultures emphasize family values such as family cohesion, mutual assistance, and close relations with extended family members^74^. However, Arab cultures are distinguished from East-Asian collectivistic cultures in multiple aspects, including geography, language, family structure^75^, expression of values^76^, and attitude toward authority^77–79^. Unjustified generalization of cultures diminishes distinguished cultural features^80^. Moreover, it can indirectly lead to the consistent exclusion of certain populations and their misrepresentation in scientific literature^81^. For example, while Eastern collectivistic cultures, such as the Indian and Sudanese, have low post-partum depression^82^, the postpartum depression rates in Arab mothers are higher than in Western cultures immediately after birth^83^. This emphasizes the importance of including Arab mothers in medical research.

In contrast to the Palestinian-Arab society, the Israeli-Jewish society has been extensively studied in the context of mother–infant relations^84–89^. The Jewish population in Israel tends to resemble Western cultures more than the Palestinian-Arab society^90,91^. Unlike the collectivist family identity of Arab culture^72,73^, the Jewish family identity has shifted towards diversity, individualism^92^, and egalitarianism^91,93,94^. Despite modernization processes seen in the Palestinian-Arab society, a cultural gap in values between the two societies is still evident^95^, with potential consequences to parenting and family relationships. For example, Arab adolescents report having a closer relationship with their parents compared to Jewish adolescents^91^, and Arab adults tend to rely on familial help to manage emotional problems, rather than professional help^96^. Along these lines, Israeli-Jewish children describe a less cohesive family structure compared to Israeli-Arab children^90^.

It is of particular interest to compare Palestinian-Arab and Jewish cultures living in Israel. Palestinian-Arab and Jewish societies are involved in a long-standing conflict on one hand, and are based on proximal geographical territories and sharing environmental impact on the other hand. Thus, this specific investigation represents a cultural comparison that goes beyond the common east vs. west or WEIRD vs. non-WEIRD studies, which can shed light on additional sources of cultural differences and similarities in affect. A few cross-cultural studies that include Palestinian-Arab mothers were conducted in Israel, in which the population comprises a relatively individualistic Jewish society (74.4%) and a more collectivistic Palestinian-Arab society (20.9%)^97^. It was demonstrated that Palestinian-Arab parents better adapt to work following the first childbirth compared to Jewish parents, as infants were cared for by a family member^98^. One previous study compared Jewish and Palestinian-Arab parent–infant dyads and found increased social gaze and active touch in Jewish dyads compared to Palestinian-Arab dyads, which showed increased proximity between parent and infant and reduced negative emotionality compared to Jewish dyads^99^. Interestingly, Jewish parents demonstrated higher levels of parental reciprocity with the infant during interaction compared to Palestinian-Arab parents^98^. With that, despite the different patterns of parent and infant behavior, levels of infant self-regulation^98^ and attachment styles^100^ were comparable, suggesting that different behavioral patterns can achieve similar developmental purposes. Synthesizing these findings points out that affective communication in the parent-infant dyad may depend on culture. Specifically, research in adults suggests that Jewish and Palestinian mothers may have distinct patterns of affective communication^101,102^. Moreover, developmental research on the impact of parents on infant development of emotion regulation^99,103–105^, points out that the cultural differences in maternal affect may underlie a culturally distinct pattern of affect regulation in Jewish and Palestinian infants.

Here we use Affect Regulation Coding System (ARCS), which captures in a dynamic way how valence and arousal are spontaneously communicated by infants and mothers during free interactions, to test whether affect communication and co-regulation show cross-cultural variation among Jewish and Palestinian-Arab families. Using ARCS, we aim to discover (1) whether there are cultural differences in the regulation of valence and arousal in Palestinian and Jewish mothers; (2) whether those mediate further cultural differences in the regulation of valence and arousal of Palestinian and Jewish infants; and (3) whether Palestinian and Jewish mothers use different strategies to regulate their infants.

## Results

### Cultural differences in mother–infant valence and arousal

**Figure 1:**
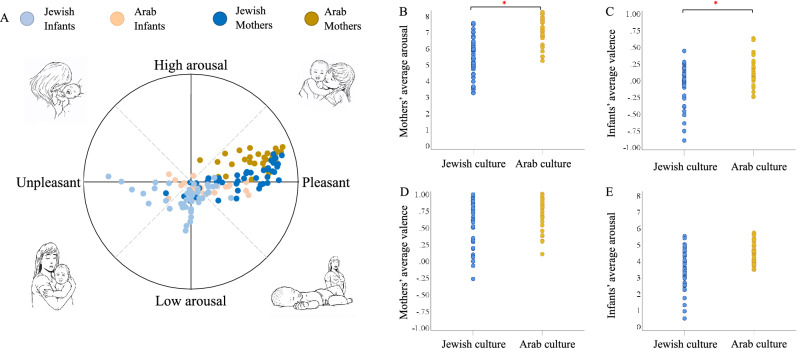
Cultural differences in valence and arousal during free mother–infant interaction. (**A**) The horizontal axis tracks the expression of valence, and the vertical axis tracks the intensity of the affective expression. Jewish participants are represented in shades of blue (dark for the mothers and light for the infants), and Palestinian-Arab participants are represented in shades of yellow (dark for the mothers and light for the infants). (**B**) Cultural differences are found in maternal arousal. (**C**) Cultural differences are found in infant valence. (**D**) Cultural differences were not found in maternal valence. (**E**) Cultural differences were not found in infant arousal.

Multivariate GLM demonstrates a statistically significant difference between cultures in Global Scores of valence and arousal during mother–infant interactions: Palestinian-Arab mothers have increased arousal compared to Jewish mothers (*F* (1, 69) = 26.068, *p* < 0.001), and Palestinian-Arab infants have more positive valence compared to Jewish infants (*F* (1, 69) = 4.561, *p* = 0.036) (Fig. 1). Specifically, Maternal arousal: Palestinian-Arab mothers have higher arousal while interacting with their infant compared to Jewish mothers (Arab: M = 6.87, SE = 0.19, 95% CI 6.8, 7.25]; Jewish: M = 5.47, SE = 0.16, 95% CI [5.16, 5.79], *p* < 0.001, 95% CI [− 1.480, − 0.495]). (C) Infant valence: Palestinian-Arab infants are tending to show increased valence when interacting with their mothers compared to Jewish infants (Arabs: M = 0.103, SE = 0.049, 95% CI [0.005, 0.201]; Jewish: M = − 0.41, SE = 0.04, 95% CI [− 0.121, 0.039]; *p* = 0.036, 95% CI [0.01, 0.279]). (D) Maternal valence: there is no significant difference in valence among Palestinian-Arab and Jewish (Arabs: M = 0.652, SE = 0.06, 95% CI [0.534, 0.770]; Jewish: M = 0.603, SE = 0.05, 95% CI [0.507, 0.700]; *p* = 0.466, 95% CI [− 0.211, 0.114]). (E) Infant arousal: there is no significant difference between Palestinian-Arab and Jewish infants in arousal when interacting with their mothers (Arabs: (M = 4.13, SE = 0.173, 95% CI [3.056, 3.719]; Jewish: M = 3.73, SE = 0.141, 95% CI [3.45, 4.02]; *p* = 0.267, 95% CI [− 0.707, 0.199]).

### Mediation analysis on the relationship of culture, infants’ valence, and mothers’ arousal

**Figure 2:**
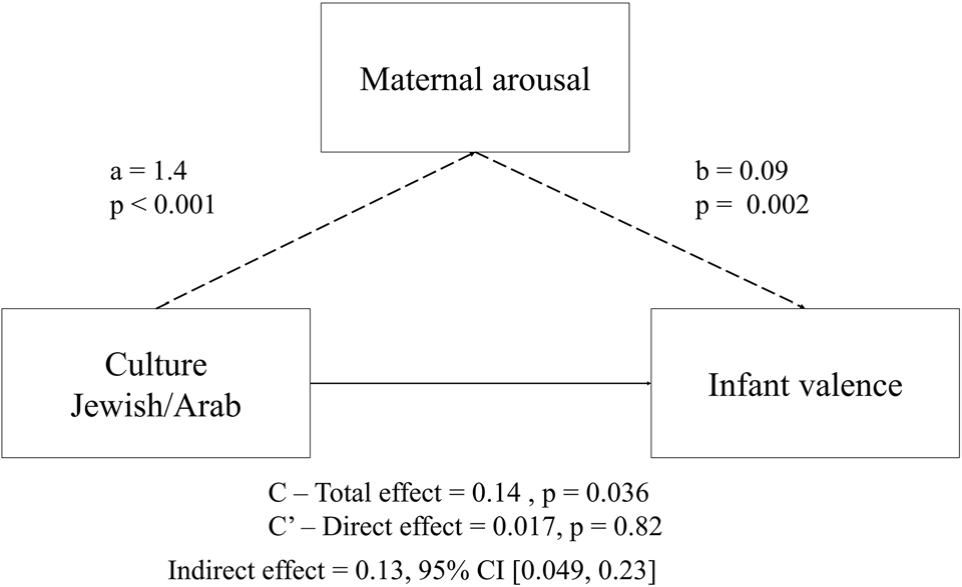
Maternal arousal mediates the association between culture and infants’ valence.

Mediation analysis demonstrates that the cultural difference in infant valence is mediated by maternal arousal (Simple Mediation Analysis: Total effect = 0.14 *p* = 0.036, Direct effect = 0.017 *p* = 0.82, Indirect effect = 0.13 95% CI [0.049, 0.23]) (Fig. 2). We also examine a mediation model with the opposite direction, where infant valence is a mediator of the cultural effects in maternal arousal (Total effect = 1.396, *p* < 0.001, Direct effect = 1.193 *p* < 0.001, Indirect effect = 0.203, 95% CI [0.035, 0.402]). In the first mediation model, the direct affect becomes insignificant when including the mediator, suggesting that the variation in infant valence is explained by maternal arousal. In the second model, the direct effect remained significant, suggesting that while some of the variation in maternal arousal is explained by the infant valence, culture also directly explains the variation in maternal arousal. Importantly, since the data are not longitudinal these mediation analyses cannot count for causality.

### Cultural differences in affect regulation

Cultural differences were not found in dynamic synchrony but were found in maternal responses during events of infant regulation and in mutual affective match.

#### No cultural differences found in mother–infant dynamic synchrony of valence and arousal

Results demonstrate individual differences in dyadic synchrony of valence and arousal in both cultures, where some mothers and infants are highly synchronized, and some are poorly synchronized. No cultural differences were found in the distributions of dynamic synchrony of valence and arousal (Valence: *t*_(71)_ =  − 0.97, *p* = 0.336, two-tailed; Jewish dyads *mean r* = 0.108, 95% CI [0.066, 0.15], Palestinian-Arab dyads *mean r* = 0.080, 95% CI [− 0.050, 0.066]. Arousal: *t*_(65)_ =  − 0.05, *p* = 0.964 two-tailed; Jewish dyads *mean r* = 0.19, 95% CI [0.143, 0.237], Palestinian-Arab dyads (*mean r* = 0.19, 95% CI [0.150, 0.230]) (Figure S2).

#### Cultural differences in maternal affect during infant affective regulation

**Figure 3:**
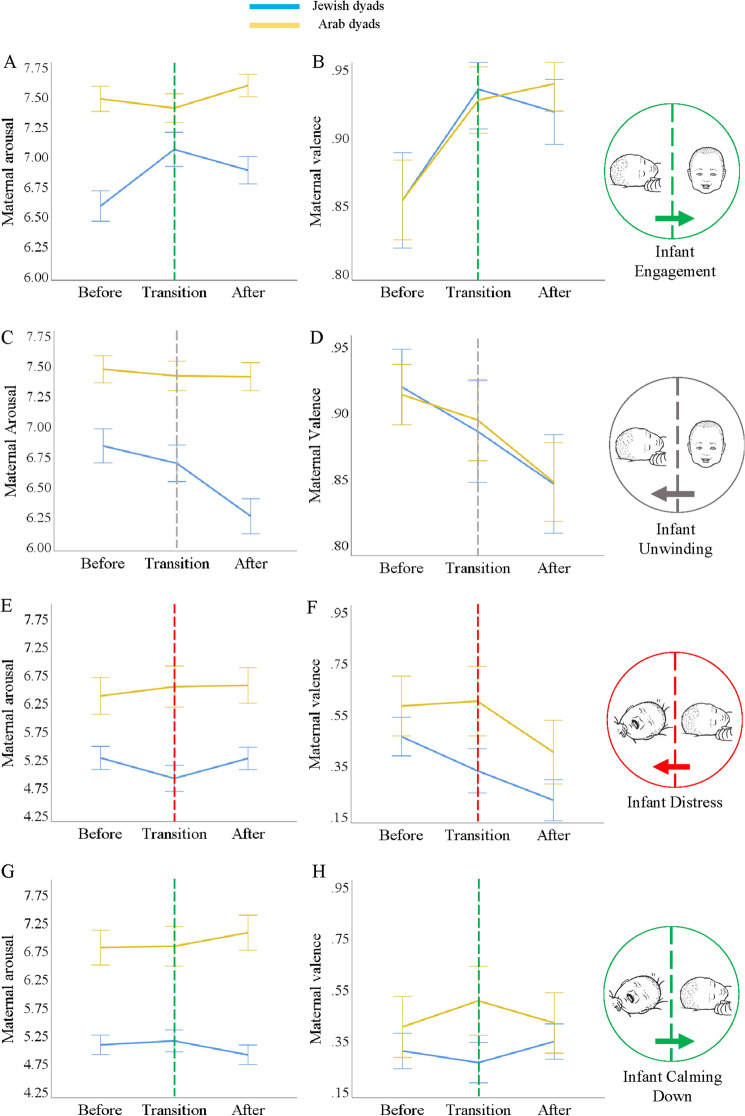
Maternal changes in valence and arousal during infant engagement, distress, unwinding, and calming down. The X-axes represent time, where multiple events of infant play or distress are temporally aligned in time at x = 0. Y-axes represent maternal social display of valence or arousal during infant play, distress, unwinding, and calming down. Error bars represent 1 standard error (SE). (**A**) Maternal arousal during infant engagement. (**B**) Maternal valence during infant engagement. (**C**) Maternal arousal during infant unwinding. (**D**) Maternal valence during infant unwinding. (**E**) Maternal arousal during infant distress. (**F**) Maternal valence during infant distress. (**G**) Maternal arousal during infant calming down. (**H**) Maternal valence during infant calming down.

Cultural differences were found in how mothers respond to events of infant regulation (Fig. 3). ARCS provides second-by-second measures of dyadic affect, which enables to assess in a dynamic way how maternal affect unfolds during specific events of infant affective regulation. Maternal responding was assessed during four types of infant regulatory events: infant engagement into play, infant unwinding, infant distress, and infant calming down. The results show that Palestinian-Arab mothers maintain higher arousal compared to Jewish mothers regardless of the infant's affect. However, Palestinian-Arab mothers maintained more positive valence compared to Jewish mothers only at times of infant distress (Fig. 3). See statistical analysis for analyses of all time points in Supplementary Table S2.

##### Maternal arousal during infant play

Palestinian-Arab mothers express elevated levels of arousal compared to Jewish mothers during events of infant engagement into play (Repeated Measure GLM, *F* = 18.25, *p* < 0.001, η2 = 0.091). While Palestinian-Arab mothers remain in high arousal throughout the infant transition into play, Jewish mothers show an increase in arousal that precedes the infant transition into play (Δ stages [before] vs [transition] =  − 2.32, p = 0.021 two-tailed, 95% CI [− 0.789, − 0.066]) (Fig. 3A).

##### Maternal valence during infant play

There is no cultural difference in maternal valence between Palestinian-Arab and Jewish dyads during infant engagement into play (Repeated Measure GLM, *F* = 0.02, *p* = 0.889, η2 = 0.00) (Fig. 3B). Palestinian-Arab and Jewish mothers show a gradual increase in positive valence that precedes the moment of infant positive transition (Palestinian-Arab mothers: Δ stages [before] vs [transition]: t = − 2.16, *p* = 0.034 two-tailed, 95% CI [− 0.153, − 0.006], Δ stages [before] vs [after]: t = − 2.32, *p* = 0.022 two-tailed, 95% CI [− 0.159, − 0.012]; Jewish mothers: Δ stages [before] vs [transition]: t = − 1.92, *p* = 0.056 two-tailed, 95% CI [− 0.147, 0.002]) (Fig. 3B).

##### Maternal arousal during infant unwinding

Palestinian-Arab mothers express elevated levels of arousal compared to Jewish mothers during events of infant unwinding (Repeated Measure GLM, F = 26.25, p < 0.001, η2 = 0.134). While Palestinian-Arab mothers remain in high arousal throughout the infant transition into neutral, Jewish mothers show a decrease in arousal that precedes the infant transition into neutral (Δ stages [before] vs [after]: t = 2.5, *p* = 0.013 two-tailed, 95% CI [0.116, 0.995]) (Fig. 3C).

##### Maternal valence during infant unwinding

There is no cultural difference in maternal valence between Palestinian-Arab and Jewish dyads (Repeated Measure GLM, F = 0.001, *p* = 0.972, η2 = 0.00.) Palestinian-Arab mothers show a gradual decrease in positive valence that precedes the moment of infant transition from positive to neutral, a similar trend is observed in Jewish mothers (Palestinian-Arab mothers: Δ stages [before] vs [after]: t = 2.15, *p* = 0.035 two-tailed, 95% CI [0.006, 0.165]; Jewish mothers: Δ stages [before] vs [after]: t = 1.38, *p* = 0. 352 two-tailed, 95% CI [− 0.019, 0.106]) (Fig. 3D).

##### Maternal arousal during infant distress

Palestinian-Arab mothers express overall elevated levels of arousal compared to Jewish mothers during events of infant distress (Repeated Measure GLM, *F* = 10.68, *p* = 0.001, η2 = 0.09). There were no significant temporal changes in maternal arousal (Fig. 3E).

##### Maternal valence during infant distress

There is no cultural difference in maternal valence between Palestinian-Arab and Jewish dyads (Repeated Measure GLM, F = 1.542, *p* = 0.217, η2 = 0.014). Jewish mothers show a gradual decrease in valence that precedes the moment of infant display of distress, a similar trend was observed in Palestinian Arab mothers (Jewish mothers: Δ stages [before] vs [after] t = 2.72, p = 0.007 two-tailed, 95% CI [0.07, 0.441]; Palestinian-Arab mothers: Δ stages [before] vs [after]t = 2.01, p = 0.11 two-tailed, 95% CI [− 0.05, 0.342]) (Fig. 3F).

##### Maternal arousal during infant calming down

Palestinian-Arab mothers express elevated levels of arousal compared to Jewish mothers during events of infant transition from negative to neutral (Repeated Measure GLM, F = 27.396, *p* < 0.001, η2 = 0.209). Palestinian-Arab mothers remain in high arousal throughout the infant transition into neutral, whereas Jewish mothers remain in low arousal throughout the infant transition to neutral. There were no significant temporal changes in maternal arousal (Fig. 3G).

##### Maternal valence during infant calming down

There is no cultural difference in maternal valence during events of infant calming down (Repeated Measure GLM, F = 1.424, *p* = 0.235, η2 = 0.014). There were no significant temporal changes in maternal valence (Fig. 3H).

#### Cultural differences in mother–infant affective matching

**Figure 4:**
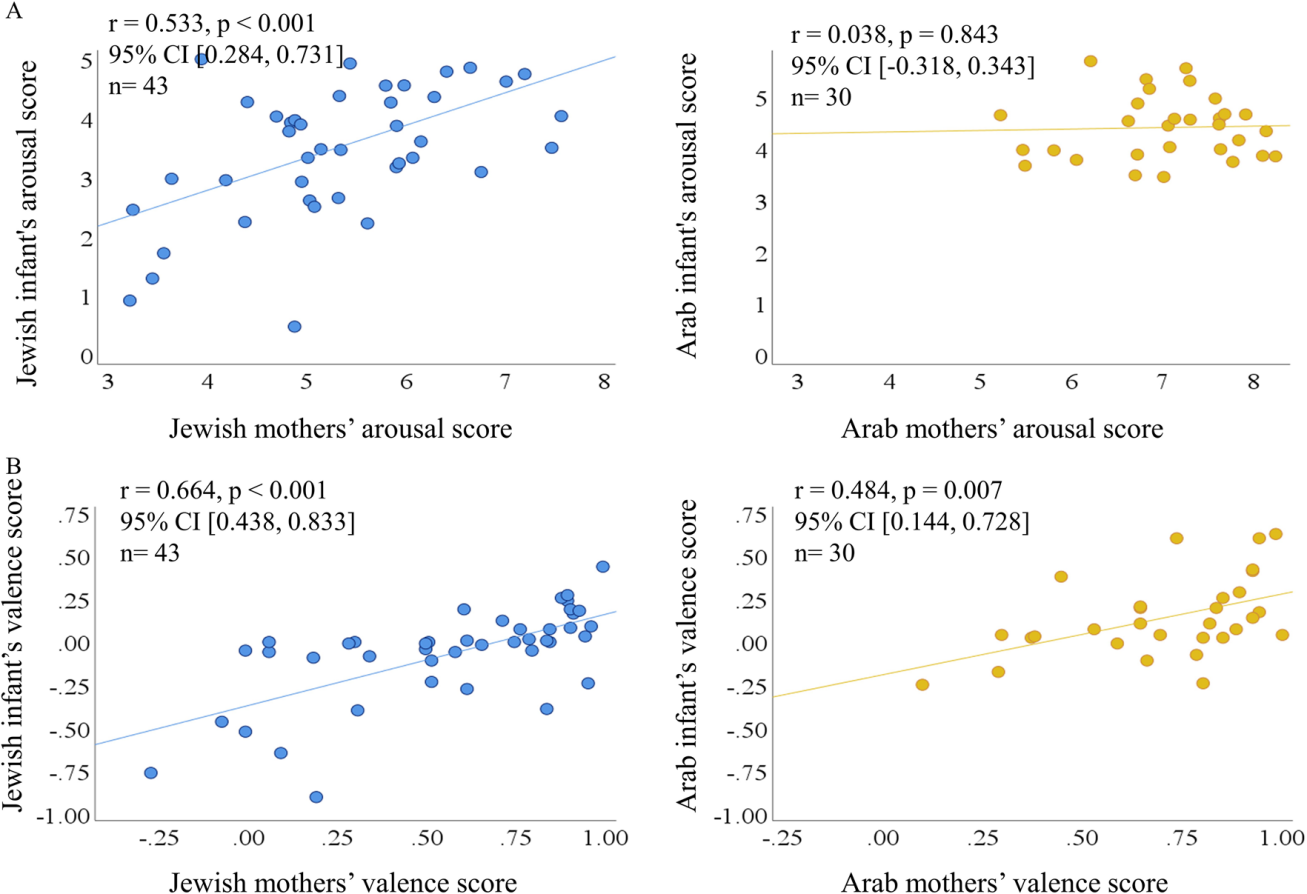
Mother–infant mutual affective match in Arab and Jewish cultures. (**A**) Mutual match between mothers and their infants in arousal only in Jewish dyads. (**B**) Mutual match between mothers and their infants in valence in Jewish and Palestinian-Arab dyads.

Both Palestinian-Arab and Jewish dyads show strong valence mutual match, while Jewish dyads are more matched in their arousal (Fig. 4). The mutual affective match is the Pearson correlation between the Global Valence or Arousal Scores of the mothers and the infants.

##### Mutual match in arousal

Mothers and infants are highly correlated in their global scores of arousal in Jewish dyads (*r* = 0.533, *p* < 0.001, 95% CI [0.284, 0.731]), and not significantly correlated in Palestinian-Arab dyads (*r* = 0.038, *p* = 0.843, 95% CI [− 0.318, 0.343]). This cultural difference is significant (*z* = 2.23, *p* = 0.026, two-tailed).

##### Mutual match in valence

Mothers and infants are highly correlated in their global scores of valence in both Jewish and Palestinian-Arab dyads (Jewish, *r* = 0.664, *p* < 0.001, 95% CI [0.438, 0.833], Palestinian-Arab, *r* = 0.484, *p* = 0.007, 95% CI [0.144, 0.728]). There is no statistical difference in valence mutual match between Arab and Jewish cultures (*z* = 1.09, *p* = 0.275, two-tailed).

## Discussion

This study demonstrates that basic features of maternal and infants’ affect during free interaction, including affect communication and co-regulation, are not universal and show cross-cultural variation. Specifically, Palestinian-Arab mothers show elevated arousal and Palestinian-Arab infants display more positive valence during free interaction compared to Jewish dyads. The levels of maternal arousal mediate the cross-cultural difference in infant valence. While Palestinian-Arab mothers maintain stable levels of high arousal, Jewish mothers show variation in their arousal levels, which co-varies with infant arousal. During times of infant distress, Palestinian-Arab mothers maintained more positive valence compared to Jewish mothers who were more matched to the infant valence. This points to a cross-cultural difference in maternal strategies for infant regulation: Palestinian-Arab mothers maintain more positive infants, while Jewish mothers maintain a better mutual affective match with their infants. This study shows that basic maternal behaviors that are often considered typical and universal manifest differently across cultures, suggesting that mother–infant interactions can serve as a mechanism via which cultural socialization of emotion can be transmitted between generations^106^.

While no cultural differences were found in dynamic synchronization across the entire interaction, ARCS enables us to zoom into discrete moments of infants’ regulatory transitions, where cultural differences do emerge. The results show that infant engagement is consistently preceded by upregulation of positive valence in Palestinian-Arab mothers, and upregulation of both valence and arousal in Jewish mothers, potentially as a strategy to engage the infant. When calming down, Jewish mothers down-regulate their arousal in attunement with the infant, unlike Palestinian-Arab mothers who maintain high arousal while their infants calm down. Moreover, unlike Jewish mothers, Palestinian-Arab mothers use positive valence to soothe their distressed infants. With that, despite the cultural specifications during positive transitions, both Palestinian-Arab and Jewish mothers are equally attuned to their infants during negative transitions (distress and unwinding). The cross-cultural commonality in the response to infant distress could reflect its immediate allostatic consequences that are relevant for survival. On the other hand, in times of infant engagement, cultural expressions become more pronounced.

The elevated arousal of the Palestinian-Arab mothers and the increased mutual affective match of the Jewish mothers can represent two strategies of infant regulation. Higher arousal was found to be related to greater infant attention to their mothers^107^. Parent-infant similarities were also found to promote joined attention, as well as the development of complex affective expression, and the ability to abstract^108^. Therefore, the high levels of arousal that were found in the Palestinian-Arab mothers and the affective match in Jewish mothers can both serve as strategies for the social regulation of infants’ attention that maintain their engagement. This suggests that while the need to maintain infants engaged and regulated is a universal feature of parental care, different regulation strategies can be applied, and those may vary cross-culturally.

Adult literature reported that Palestinian-Arab and Jewish cultures show differential patterns of affective communication^101,102^. Tracing cultural differences previously reported in adults to the early mother–infant bond can advance our understanding of socialization of affect and emotion. The study demonstrates that Palestinian-Arab infants show more positive affect while interacting with their mothers and that this cross-cultural effect is mediated by maternal arousal. This result is consistent with previous cross-cultural studies that found that Arab infants showed fewer negative affect compared to Jewish infants^99^. Collectivist cultures associate positive feelings with social harmony^109^, which may elicit maintained positivity in Arab culture. Accordingly, the high arousal of the Palestinian-Arab mothers in our study may reflect an effort to keep the infant engaged in a positive state. Together, these findings in adults and infants point out that self-regulation can be rooted in cultural norms, and sculpted at an early age via mother–infant interactions^110^. By responding to the infant's affective signals, mothers and other caregivers provide immediate regulation but also help the infant to become more independent in self-regulation^39,111–114^. Indeed, from an early age, infants learn to adjust their response to their caregiver's behavior^115^, and thus parents can shape their infant's behavior according to their cultural patterns. Therefore, the culture-specific pattern of both mothers’ and infants’ behaviors can represent a behavioral mechanism for the cross-cultural transmission of behavioral norms.

Despite cultural differences, a similar percentage of mothers chose to interact in a face-to-face position (87% of Palestinian-Arab mothers, and 79% of Jewish mothers). Previous research has demonstrated that face-to-face positions are common in individualist cultures, which are characterized by active eye-gaze transactions. In collectivist cultures, there is more physical proximity and continuous physical contact with less direct eye-gaze interactions^99,116^. This suggests that the individualist/collectivist distinction may not apply in comparing Jewish to Palestinian-Arabs in Israel and emphasizes the importance of studying specific cultures. Another cultural similarity was demonstrated in dynamic synchrony, where no cultural differences emerged, as a similar variance of individual differences in dyadic synchrony of valence and arousal was observed in both cultures. Such distribution of individual differences in mother–infant synchrony aligns with previous research^117,118^, demonstrating the reliability of ARCS measurements. This supports previous research proposing that the tendency to synchronize in attunement with a partner during interaction is a universal phenomenon^63^. Future cross-cultural research is needed to determine this in many individuals across multiple cultures.

This research holds some limitations. The extent of the compatibility of experimental constructs to both cultures, such as the behavioral coding of valence and arousal, may vary as specific measurements can be more prominent in certain cultural settings. Moreover, research constructs can be defined differently, depending on culture, and impact the results of the study^115^. However, importantly, the method used in this research was developed by Palestinian-Arab and Jewish scientists on a sample of both Palestinian-Arab and Jewish populations. Another limitation is that the developmental consequences of such cultural differences were not assessed in this study. Previous cross-cultural longitudinal research found that similar maternal behaviors, such as maternal intrusiveness in infancy, can lead to different outcomes across cultures^119^. In this study, the statistical control over infant ages interferes with assessing the developmental trajectory of cultural differences. Thus, we are unable to determine whether Jewish and Palestinian-Arabs mothers adapt differently to their infants’ affective development. This possibility is supported by the second mediation model showing that infant valence partly mediates the cultural differences in maternal arousal. Given prior literature suggesting that early-life socialization is a bidirectional process^120,121^, future longitudinal research is needed to determine the developmental outcomes of the early cross-cultural differences found here on both infants and parents, whether they remain consistent, and how they affect child development.

In sum, cultural differences in affect communication and regulation are evident in early life. Such cross-cultural differences indicate that basic features of emotion that are often considered universal, are differentially represented in different cultures. Most studies of cultural differences between mothers and children focus on comparing east with west, or WEIRD with Non-WEIRD cultures. Thus, including the Palestinian-Arab culture that is underrepresented in medical and psychological research, and comparing it to the geographically adjacent Jewish culture, can provide novel evidence on the origins of cultural similarities and differences.

## Methods

### Participants

Seventy-three mother–infant dyads participated in the study, 30 mothers who identified as Palestinian-Arabs (13 female infants and 17 males) and 43 mothers who identified as Jewish (17 female infants, 26 males), all of them hold Israeli citizenship. Infants’ ages ranged between 2.5 and 48 weeks and mothers’ ages ranged between 22 and 47 years. The sample size was determined with power analysis calculated for multivariate General Linear Model (GLM) using G*Power software^122^, given an effect size of 0.4, and power of 0.8 for comparing mother–infant interactions within two different cultures, relying on data from our previous research on mother–infant bonding behavior^123,124^. Palestinian-Arab and Jewish families were matched in demographic variables including education (Mean Jewish years of education = 16.88 [SD = 1.48], Mean Palestinian-Arab years of education = 15.67 [SD = 1.83]), income level (Jewish income score = 3.3 (SD = 1.66), Palestinian-Arab income score = 3.23 (SD = 1.73) and marital status (all mothers were married). Group differences were found in infant age (Mean Jewish infant age = 13.5 weeks; Mean Palestinian-Arab infant age = 24 weeks) and the number of children in the family (Mean Jewish = 1.6, Mean Palestinian-Arab = 2.4), accordingly, all the statistical analyses that compare Jewish to Arab cultures control for variability explained by these variables, unless otherwise stated. The Institution Ethics Committee of the Hebrew University approved the study, the research protocol was performed in accordance with ethics guidelines, and all the mothers signed informed consent before participation and were remunerated for their participation. And filled out questionnaires in the presence of the experimenter, so that it was possible to get used to his presence at this time.

### Procedure

Mothers were recruited for the study via social media ads. Mothers were not informed about the research questions before participation to optimize the ecological validity of the experimental procedure. Participation included questionnaires and a video recording of a 2-min free interaction with the infant. Mothers spend up to twenty minutes participating and were remunerated. Videos were captured in the families’ homes or their local community center. Trained research personnel filmed the interaction while in the room, based on our previous research^33,34,42,125^. Mothers and infants had time to adjust to the research personnel before filming. Interactions were filmed using one camera, while research personnel confirmed that the face and body of both dyadic partners are captured in the frame throughout the interaction^33,34,42,125^. Seconds in which faces were not captured were removed from the analysis (Infants’ face: mean = 1.47 s, STD = 4.15, median = 0; Mothers’ face: mean = 0.59 s, STD = 2.04, median = 0). There were no missing data for participants’ bodies. It is acknowledged despite the relatively naturalistic out-of-lab setting of the filming, the presence of the experimenter may affect the spontaneous behavior of mothers and infants. Mothers were instructed to freely interact with their infants as they usually do, without any restrictions, specific toys, or guidelines. This setting was found to be highly representative of natural mother–infant interactions^126,127^, as it allows each mother to determine her own setup and interaction choices and tendencies with no constraints. Interestingly, 87% of Palestinian-Arab and 79% of Jewish mothers chose to interact in a face-to-face position.

Capturing 2-min interactions optimizes the trade-off between the high validity of the data on one hand and minimal intrusion to post-partum women and their infants on the other hand. Accordingly, an experimental setup of 2-min mother–infant interactions is commonly applied^38,123–125,128–135^. While short and thus susceptible to being affected by non-representative events, this data is rich in spontaneous interactive behaviors, providing a multi-dimensional matrix of 120 repeated data points per each behavioral variable for each dyadic partner. Such a matrix is highly detailed and more ecologically valid compared to questionnaires or lab-based tasks. To confirm the reliability of each measurement across the interaction, a Kolmogorov–Smirnov test was applied (Supplementary analysis 2). The free mother–infant interactions were then imported to the lab and behaviorally coded and analyzed using ARCS by trained personnel.

### Behavioral analyses

Trained coders coded behavior in the videos of the mothers and infants interacting. Coders tracked concrete behaviors that represent the expression of valence and arousal. The coders tracked the second-by-second behavioral display of valence and arousal, separately for each ARCS scale for each dyadic partner, throughout the interaction (see ARCS scales in Table 1). Coders observed the videos of the interactions on Windows Media Player and stopped after each second to code the specific ARCS scale. Each second during the interaction video was given a score per each ARCS scale. The coding is then quantified either as global scores, i.e., average across 120 s (in Figs. 1, 2, 4), or used in temporal analyses as second-by-second value (Fig. 3 and dynamic synchrony analysis).

**Table 1:** ARCS—affect regulation coding system.

Axis	Behavioral parameter	Codes
Valence	Facial expressions	Negative (for example crying, angry face, curving the lips downwards) Neutral (the absence of signs of negative or positive valence) Positive (for example smiling, laughing, curling the lips upwards)
Arousal	1. Physical effort—bodily movement	(− 2) Sleeping (− 1) Falling asleep (0) Calm with no movement (1) Subtle movement (for example, gentle hand movements, petting an object) (2) Significant movement (for example, large movements with the hands, lifting objects) (3) Intensive movement (for example, a movement that involves the whole body, walking)
	2. Agency—gaze and attention	(0) Unfocused look (for example staring into space (1) Directed gaze (for example, the gaze is focused on a specific object (2) Switching gaze (for example, the gaze is moving from one object to another)
	3. Vocalization—pitch and intensity	Mothers: (0) No vocalization (for example the mother is silence) (1) Low pitch (for example the mother whispered) (2) Adult speech (for example the mother talk regularly) (3) High pitch intensity (for example the mother talking in a high pitch, the mother is talking in a childish way or storytelling) Infants: (0) The infant is silent (1) The infant makes small noises or weak syllables (2) The infant makes a sound at regular volume (3) The infant cries, laughs or shouts
	4. Expression intensity—the amplitude of the facial expression	(0) No facial effort (no facial expression) (1) Vocalization with no expression) (2) Mild expression (for example, a weak smile) (3) Strong expression (for example, an expression of surprise, a big smile, a sullen expression)

#### ARCS—affect regulation coding system

##### Valence

Valence codes included scores of 1 (positive), 0 (neutral), or − 1 (negative), based on the pleasantness of the facial expression.

##### Arousal

The arousal score is based on coding the behavioral expressions of four continuous sub-scales: (1) physical effort (measured with body motion)^136^; (2) agency (measured with gaze and attention)^137,138^; (3) vocalizations (measured with pitch and intensity of the vocalization)^139^; and (4) expression intensity (measured with by the amplitude of the facial expression). More information on how to apply ARCS will be freely provided by the corresponding author upon request.

#### Compute regulation scores of mothers and infants during interaction

After the initial coding of infants’ and mothers’ valence and arousal, we used MATLAB R2016a (The MathWorks, Natick, MA) and Python (version 3.7) to plot the scores of each subject on a bi-dimensional space of valence and arousal^140^, and computed score for General Valence (Eq. 1), General Arousal (Eq. 2), and dynamic measurements of regulation during the interaction (Fig. 1).

Equation (1) :General Valence Score: across 120 s of the interaction (N).

$$\overline{v }=\frac{\sum {v}_{i}}{N}$$

Equation (2): General Arousal Score: relies on a summary of four subscales: physical effort, agency, vocalization, and expression intensity (Table 1), which were coded separately. Then, the four sub-scales were normalized on a scale of 0–10 and averaged per each second. Last, for each participant, the multi-scale score is averaged across 120 s (N).

$$\overline{a }=\frac{\sum {a}_{i}^{effort}+{a}_{i}^{agency}+{a}_{i}^{vocalization}+{a}_{i}^{expression intensity }}{N}$$

#### Inter-rater reliability

For each ARCS variable, at least 8% of videos were coded by multiple coders to validate the reliability of ARCS coding. The reliability was calculated for the entire interaction. Krippendorff’s alpha test was used to estimate the inter-rater reliability between coders, given its accuracy in estimating the level of agreement between raters for categorical variables with multiple levels^141^. The inter-rater reliability scores for maternal behavior: Krippendorff’s α = 0.85; and for infant behaviors: Krippendorff’s α = 0.89, which is considered moderate to high^141^ (see Supplementary Table S1 for reliability scores of each variable).

#### Validity of the coding scheme

The empirical implementation of the axes relies on previous literature, showing that the behavioral display of physical effort^136^, agency and attention^137,138^, and vocalization^139^ are related to levels of arousal. It also relies on previous methods that code affect or valence during interactions^42,125^. ARCS is rooted in the theoretical framework of the affective circumplex by James Russel^140^ and sets to map the interplay of valence and arousal on a bi-dimensional space in two partners simultaneously, to trace dyadic patterns of co-regulation. ARCS analyses demonstrate expected phenomena, including expected variation in parent-infant dynamic synchrony^142^, and arousal responsivity to salient stimuli^143^.

### Test the cultural differences in arousal, valence, and co-regulation

#### Test the cultural differences of arousal and valence in mothers and infants

The General Valence and Arousal scores of mothers and infants in each cultural group were mapped on a bi-dimensional space of valence and arousal^144^ (Palestinian-Arab = 30, Jewish = 43). We applied a multivariate GLM to test the group differences in general valence and arousal scores between Palestinian-Arab and Jewish mothers and infants (Fig. 1).

#### Test if maternal affect mediates the cross-cultural difference in infants’ affect

We used a Simple Mediation Model with 1000 bootstrap samples to calculate the direct effect of culture on infant affect, and whether the cross-cultural differences in infant affect are mediated by maternal affect, or whether the cross-cultural differences in maternal affect are mediated by infant affect. Mediational analysis was performed via Hayes's (2013) “PROCESS" macro, model 4, v2.16 mediation script for SPSS (Fig. 2).

#### Test for cultural differences in affect regulation

Cultural differences were tested in three dyadic patterns that reflect co-regulation: (a) dynamic synchrony; (b) maternal responding during events of infant regulation; (c) mutual affective match.Dynamic synchrony of valence and arousal

##### Dynamic synchrony of valence and arousal

To evaluate the cross-cultural difference in dynamic synchrony between mothers and infants, we calculated the Pearson correlations for arousal and Cramer’s coefficient for valence^145^ across the 120 s of the interaction. Scores were calculated in each culture separately, and the distributions of synchrony scores were compared cross-culturally.

##### Maternal responding during events of infant regulation: engagement, distress, unwinding, and calming down

In each type of infant event, maternal valence and arousal were averaged across all participants for a given second and compared cross-culturally with repeated measure GLM (Fig. 3). Post hoc LSD assessed the simple effects in each culture (Supplementary Table S2).

*Infant engagement* Events of infant engagement were defined as transitioning from neutral to positive valence. We located all events where infants transitioned from neutral to positive valence across all the free interaction videos aligned in time (187 events across 48 mother–infant interactions: Palestinian Arabs = 109, Jews = 78). We then calculated the average scores of maternal valence and arousal 3 s before and 3 s after the infant transition to dynamically map maternal affect during events of infant engagement.

*Infant unwinding* Events of infant unwind were defined as events where infants transitioned from positive to neutral valence: we located all events where infants transitioned from positive to neutral valence across all of the free interaction videos aligned in time (174 events across 47 mother–infant interactions: Palestinian-Arabs = 105, Jews = 69). We then calculated the average scores of maternal valence and arousal 3 s before and 3 s after the infant transition to dynamically map maternal affect during events of infant unwinding,

*Infant distress* Events of infant distress were defined as events where infants transitioned from neutral to negative valence. We located all events where infants transitioned from neutral to negative valence across all the free interaction videos aligned in time (112 events across 38 mother–infant interactions: Palestinian Arabs = 40, Jews = 72). We then calculated the average scores of maternal valence and arousal 3 s before and 3 s after the infant transition to dynamically map maternal affect during events of infant distress.

*Infant calming down* Events of infant calming down were defined as events where infants transitioned from negative to neutral valence: We located all events where infants transitioned from negative to neutral valence across all the free interaction videos aligned in time (108 events across 37 mother–infant interactions: Palestinian-Arabs = 32, Jews = 76). We then calculated the average scores of maternal valence and arousal 3 s before and 3 s after the infant transition to dynamically map maternal affect during events of the infant calming down.

##### Mutual affective match between mothers and infants

In addition to dynamic patterns of co-regulation, we also assessed the cross-cultural difference in *Mutual Affective Match* between mothers and infants. The mutual affective match was calculated as the Pearson correlation between the Global Scores of the mother and her infant, separately in each culture. Then, the r values were transformed using Fisher r-to-z and statistically compared cross-culturally (Fig. 4).

In all analyses, we computed the bootstrapped confidence intervals (CI). All analyses were corrected for multiple hypotheses testing using Bonferroni correction and were controlled for infant age and the number of children in the family.

## Supplementary Information

Supplementary Information.

## Data Availability

All data was uploaded to osf.io and can become publicly available upon request after publication.
