# Supplementary material for: Evidence for cultural differences in affect during mother–infant interactions
Source: Sci Rep. 2023 Mar 24;13:4831. doi: 10.1038/s41598-023-31907-y (PMC10039016; doi:10.1038/s41598-023-31907-y)
Supplement: Supplementary file 1 — Supplementary Information. [file 41598_2023_31907_MOESM1_ESM.docx]

**Evidence for cultural differences in affect during mother-infant interactions**

Miada Abu Salih*^1^, Maayan Abargil*^1^, Saja Badarne^1^, Nathalie klein-Selle^2^, Merav Irani^1^, and Shir Atzil^1^

^1^Department of Psychology, The Hebrew University of Jerusalem, Mount Scopus, Jerusalem, Israel

^2^ Criminology Department, Bar Ilan University, Ramat Gan, Israel

Please address correspondence to: Shir Atzil, [shir.atzil@mail.huji.ac.il](mailto:shir.atzil@mail.huji.ac.il)

**Supplementary Results**

**Supplementary Table S1. The average inter-rater reliability between coders, using Krippendorff’s alpha test.**

|  | | **Parents** | **Infants** |
| --- | --- | --- | --- |
| **Valence** | | α = 0.81 | α = 0.90 |
| **Arousal** | Physical Effort | α = 0.86 | α = 0.88 |
|  | Agency | α = 0.87 | α = 0.91 |
|  | Vocalization | α = 0.86 | α = 0.92 |
|  | Expression Intensity | α = 0.85 | α = 0.82 |

**Supplementary Table S2: Maternal changes in valence and arousal during infant engagement, unwinding, distress, and calm down.** Statistical analysis for all time points Palestinian-Arab and Jewish dyads.

| maternal arousal infant engagement | before-transition | before-after | transition-after | n (events) |
| --- | --- | --- | --- | --- |
| Jewish | Δ = -0.427 ; p = 0.021 | Δ = -0.296 ; p = 0.109 | Δ = 0.132 ; p = 0.474 | 78 |
| Arab | Δ = 0.045 ; p = 0.771 | Δ = -0.115 ; p = 0.454 | Δ = -0.159 ; p = 0.299 | 109 |
| maternal valence infant engagement |  |  |  |  |
| Jewish | Δ = -0.073 ; p = 0.056 | Δ = -0.64 ; p = 0.091 | Δ = 0.009; p = 0.821 | 78 |
| Arab | Δ = -0.080; p = 0.034 | Δ = -0.086 ; p = 0.022 | Δ = -0.006 ; p = 0.870 | 109 |
| maternal arousal infant unwinding |  |  |  |  |
| Jewish | Δ = 0.159 ; p = 0.476 | Δ = 0.556 ; p = 0.013 | Δ = 0.397 ; p = 0.077 | 69 |
| Arab | Δ = 0.045 ; p = 0.773 | Δ = 0.078 ; p = 0.616 | Δ = 0.033 ; p = 0.832 | 105 |
| maternal valence infant unwinding |  |  |  |  |
| Jewish | Δ = 0.010 ; p = 0.836 | Δ = 0.043 ; p = 0.352 | Δ = 0.034 ; p = 0.469 | 69 |
| Arab | Δ = 0.035 ; p = 0.389 | Δ = 0.086 ; p = 0.035 | Δ = 0.051 ; p = 0.210 | 105 |
| maternal arousal infant distress |  |  |  |  |
| Jewish | Δ = 0.318 ; p = 0.177 | Δ = 1.582e-15; p = 1.000 | Δ = -0.318 ; p = 0.177 | 72 |
| Arab | Δ = 0.093 ; p = 0.771 | Δ = 7.265e-15; p = 1.000 | Δ = -0.093 ; p = 0.771 | 40 |
| maternal valence infant distress |  |  |  |  |
| Jewish | Δ = 0.091 ; p = 0.333 | Δ = 0.256 ; p = 0.007 | Δ = 0.164 ; p = 0.082 | 72 |
| Arab | Δ = 0.060 ; p = 0.574 | Δ = 0.171 ; p = 0.110 | Δ = 0.111 ; p = 0.298 | 40 |
| maternal arousal infant calming down |  |  |  |  |
| Jewish | Δ = -0.098 ; p = 0.677 | Δ = 0.106 ; p = 0.653 | Δ = 0.204 ; p = 0.386 | 76 |
| Arab | Δ = 0.043 ; p = 0.876 | Δ = -0.096 ; p = 0.725 | Δ = -0.139 ; p = 0.611 | 32 |
| maternal Valene infant calming down |  |  |  |  |
| Jewish | Δ = 0.018 ; p = 0.853 | Δ = -0.035 ; p = 0.853 | Δ = -0.053 ; p = 0.579 | 76 |
| Arab | Δ = -0.062 ; p = 0.595 | Δ = -0.042 ; p = 0.723 | Δ = 0.021 ; p = 0.859 | 32 |

**Cultural differences of arousal and valence in an aged-matched sub-group**

To account for potential age effects without statistically controlling for infant age, we conducted cross-cultural analyses in age-matched sub-groups in our sample (Palestinian-Arabs = 20, Jewish = 20, ages; Arabs age range = 4 – 32 and an average of 18.1 weeks, Jewish age range = 4-31.5 with an average of 17.7 weeks). A multivariate GLM was applied in Palestinian-Arab and Jewish mothers and infants, to test the cultural differences in arousal and valence in mothers and infants.


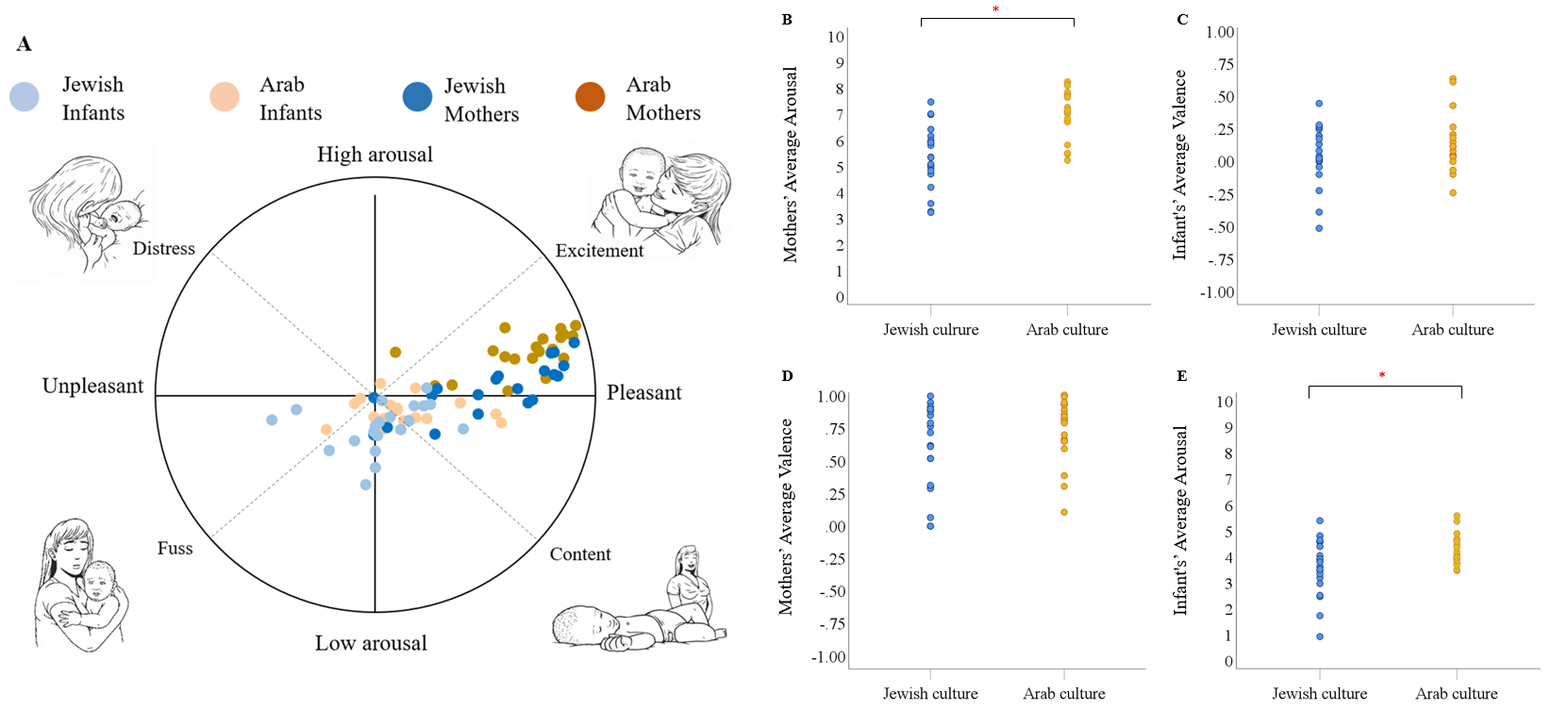


**Figure S1.** **Arab-Jewish cultural differences in valence and arousal during free mother-infant interaction in age-matched groups.** (A) Each participant receives a score of valence and arousal, which are graphically positioned on a two-dimensional space. The horizontal axis tracks the expression of valence, and the vertical axis tracks the intensity of the affective expression. (B) Maternal arousal: Palestinian-Arab mothers have higher arousal while interacting with their infant compared to Jewish mothers (Arab: M = 6.993, SE = 0.238, 95% CI [6.511, 7.475]; Jewish: M = 5.349, SE = 0.238, 95% CI [4.867, 5.832], p < 0.001, 95% CI [-2.325, -0.961]). (C) Infant valence: there is no significant difference in valence among Palestinian-Arab and Jewish (Arabs: M = 0.133, SE = 0.049, 95% CI [0.033, 0.233]; Jewish: M = 0.025, SE = 0.049, 95% CI [-0.075, 0.125]; p = 0.132, 95% CI [-0.249, 0.034]). (D) Maternal valence: there is no significant difference in valence among Palestinian-Arab and Jewish (Arabs: M = 0.735, SE = 0.065, 95% CI [0.604, 0.866]; Jewish: M = 0.588, SE = 0.065, 95% CI [0.457, 0.72]; p = 0.118, 95% CI [-0.332, 0.039]). (E) Infant arousal: There is no significant difference between Palestinian-Arab and Jewish infants in arousal when interacting with their mothers (Arabs: (M = 4.233, SE = 0.195, 95% CI [3.839, 4.627]; Jewish: M = 3.578, SE = 0.195, 95% CI [3.184, 3.972]; p = 0.022, 95% CI [-1.212, -0.098]).

**Assessing cultural differences in valence and arousal without controlling for infant age**

The results show: **Maternal arousal**: Palestinian-Arab mothers have higher arousal while interacting with their infant compared to Jewish mothers (Arab: M = 7.037, SE = 0.192, 95% CI [6.655, 7.42]; Jewish: M = 5.355, SE = 0.159, 95% CI [5.039, 5.671], p < 0.001, 95% CI [ -2.192, -1.173]). **Infant valence**: Palestinian-Arab infants are showing more positive valence when interacting with their mothers compared to Jewish infants (Arabs: M = 0.147, SE = 0.049, 95% CI [0.05, 0.245]; Jewish: M = -0.072, SE = 0.041, 95% CI [-0.153, 0.009]; p = 0.001, 95% CI [-0.350, -0.089]). **Maternal valence:** there is no significant difference in valence among Palestinian-Arab and Jewish (Arabs: M = 0.695, SE = 0.058, 95% CI [0.579, 0.81]; Jewish: M = 0.573, SE = 0.058, 95% CI [0.579, 0.81]; p = 0.119, 95% CI [-0.276, 0.032]). **Infant arousal**: Palestinian-Arab infants are showing higher arousal when interacting with their mothers compared to Jewish infants (Arabs: M = 4.367, SE = 0.184, 95% CI [4,4.734]; Jewish: M = 3.571, SE = 0.152, 95% CI [3.268, 3.874]; p = 0.002, 95% CI [-1.285, -0.307]).

**Individual differences in dynamic synchrony across cultures**

Pearson correlation was used to examine dynamic synchrony in arousal, and Cramer's coefficient of association for dynamic synchrony in Valence^113^. The distribution of individual differences in dynamic arousal synchrony was not statistically different between cultures (t (71) = -0.97, p = 0.336, two-tailed, Jewish dyads: mean r = 0.108, 95% CI [0.066, 0.15]; Palestinian-Arab dyads: mean r = 0.080, 95% CI [-0.050, 0.066]). The distribution of individual differences in dynamic valence synchrony was not statistically different between cultures (t (65) = -0.05, *p* = 0.964 two-tailed, Jewish dyads: mean r = 0.19, 95% CI [0.143, 0.237]; Palestinian-Arab dyads: mean r = 0.19, 95% CI [0.150, 0.230]).


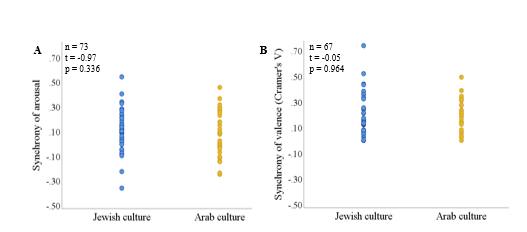


**Figure S2: Dynamic Synchrony in valence and arousal.** Arousal synchrony is calculated with Pearson correlation. Valence synchrony is calculated with Cramer's V correlation.

**Confirming the consistency of each measurement across the interaction**

We tested the distribution of each behavioral measurement in two random bins of 60 seconds. Kolmogorov-Smirnov test confirmed the similarity of the two distributions of each measurement for each dyad, supporting the reliability of these measures (Infants average arousal score: *K-S*= 0.551, *p*=0.922; Infants' valence *K-S*= 0.521, *p*=0.949; Parents average arousal score *K-S*= 1.183, *p*=0.122; Infants' valence *K-S*= 0.417, *p*=0.995).
